# Rheological Impact of GBT1118 Cessation in a Sickle Mouse Model

**DOI:** 10.3389/fphys.2021.742784

**Published:** 2021-09-24

**Authors:** Celeste K. Kanne, Danitza Nebor, Mira Pochron, Donna Oksenberg, Vivien A. Sheehan

**Affiliations:** ^1^Aflac Cancer & Blood Disorders Center Children’s Healthcare of Atlanta, School of Medicine, Emory University, Atlanta, GA, United States; ^2^Department of Pediatrics, Section of Hematology/Oncology, Baylor College of Medicine, Houston, TX, United States; ^3^Global Blood Therapeutics, South, San Francisco, CA, United States

**Keywords:** voxelotor, GBT1118, viscosity, deformability, red cell, sickle cell disease, hemorheology, rheological biomarkers

## Abstract

In sickle cell disease (SCD), higher whole blood viscosity is a risk factor for vaso-occlusive crisis, avascular necrosis, and proliferative retinopathy. Blood viscosity is strongly impacted by hemoglobin (Hb) levels and red blood cell (RBC) deformability. Voxelotor is a hemoglobin S (HbS) polymerization inhibitor with anti-sickling properties that increases the Hb affinity for oxygen, thereby reducing HbS polymerization. In clinical trials, voxelotor increased Hb by an average of 1g/dl, creating concern that this rise in Hb could increase viscosity, particularly when the drug was cleared. To investigate this potential rebound hyperviscosity effect, we treated SCD mice with GBT1118, a voxelotor analog, and stopped the treatment to determine the effect on blood viscosity and RBC deformability under a range of oxygen concentrations. GBT1118 treatment increased Hb, improved RBC deformability by increasing the elongation index under normoxic (EI_max_) and hypoxic conditions (EI_min_), and decreased the point of sickling (PoS) without increasing blood viscosity. The anti-sickling effects and improvement of RBC deformability balanced the effect of increased Hb such that there was no increase in blood viscosity. Forty-eight hours after ceasing GBT1118, Hb declined from the rise induced by treatment, viscosity did not increase, and EI_min_ remained elevated compared to control animals. Hb and PoS were not different from control animals, suggesting a return to native oxygen affinity and clearance of the drug. RBC deformability did not return to baseline, suggesting some residual rheological improvement. These data suggest that concerns regarding viscosity rise above pre-treatment levels upon sudden cessation of voxelotor are not warranted.

## Introduction

Sickle cell disease (SCD) is an autosomal recessive genetic disorder caused by a point mutation in the hemoglobin beta gene that results in the replacement of the hydrophilic glutamic acid residue with hydrophobic valine (Inusa et al., 2019). The abnormal hemoglobin S (HbS) polymerizes upon deoxygenation, distorting the red blood cell (RBC) into a rigid sickle shape. An oxygenated sickle RBC (sRBC) is less flexible, or deformable, than that of a normal individual; a deoxygenated sRBC is even less deformable (Reid and Obi, 1982; Nash et al., 1984). Over time, repetitive sickling and unsickling as a result of cycles of deoxygenation and reoxygenation as the sRBC travels through the body may lead to a permanent decrease in sRBC deformability, associated with membrane loss and dehydration (Marzouki and Khoja, 2003; Brugnara, 2018). Previous studies have shown that recurring sickling events weaken sRBCs by increasing oxidative damage, damage to the cell membrane, and consequent fragility, increasing the rate of hemolysis (Presley et al., 2008; Mohanty et al., 2014; Kato et al., 2017). As a result, individuals with SCD have decreased RBC survival and reduced Hb concentration, which impairs oxygen delivery. As sRBCs travel through the microvasculature, they display increased adhesion to the endothelium, cause obstruction, ischemia and vascular damage leading to pain crises, organ damage, and consequent early mortality (Platt et al., 1994; Gladwin and Sachdev, 2012; Manwani and Frenette, 2013; Mehari et al., 2013; Nouraie et al., 2013; Kucukal et al., 2020).

Whole blood rheology is markedly abnormal in SCD blood, with several defects in the RBC and plasma (Baskurt and Meiselman, 2003; Connes et al., 2014, 2016; Li et al., 2017). sRBC are often dehydrated (Marzouki and Khoja, 2003; Brugnara, 2018). These dense RBCs (DRBCs) are marked by high Hb amount relative to mean cell volume, are poorly deformable, and increase whole blood viscosity (Bartolucci et al., 2012; Nader et al., 2019). A high concentration of HbS also potentiates polymerization, leading to sickling. In individuals with SCD, viscosity rises more rapidly with a rise in hematocrit (Hct) than in a normal individual. For a given Hct, SCD blood is very viscous in deoxygenated conditions due to HbS sickling: An individual with SCD with a Hct of 21% would have a whole blood viscosity comparable to that of a normal individual with a Hct of 45% (Li et al., 2016). The hematocrit-to-viscosity ratio (HVR), a measure of the oxygen carrying capacity of the blood, is typically lower in individuals with SCD than normal individuals, as the denominator rises more rapidly than the numerator.

Conventional lab tests provide information on Hb levels, reticulocyte count (the number of immature RBCs), and markers of hemolysis, such as lactate dehydrogenase and unconjugated bilirubin. One can infer that low Hb and high markers of hemolysis suggest poor quality RBCs with a shortened life span. However, there are now devices that provide rheological biomarkers with more detailed, specific information on RBC quality (Lu et al., 2020). Particularly in red cell disorders like SCD, these biomarkers provide essential information on RBC function that can add to our understanding of the effects of novel therapies. Oxygen gradient ektacytometry provides measurements of RBC deformability in the oxygenated and deoxygenated state, and identifies the pO_2_ at which the first RBCs begin to sickle, termed point of sickling (PoS; Rab et al., 2019). The greatest RBC deformability (EI_max_) corresponds with fully oxygenated RBCs with the least amount of HbS polymerization. The poorest RBC deformability (EI_min_) corresponds with low oxygen conditions with the greatest amount of HbS polymerization. These three markers vary between individuals and therapies. In treating SCD, Hb quality produced is extremely important, not just amount. Therapies that increase the amount of Hb or number of RBCs without improving the quality may increase viscosity and increase risk of clinical events associated with viscosity, such as pain crises, avascular necrosis, and proliferative retinopathy (Richardson et al., 1976; Hernigou et al., 2006; Lemaire et al., 2013; Abdalla Elsayed et al., 2019; Itzep et al., 2019). It is therefore essential to evaluate multiple aspects of red cell rheology.

Voxelotor is a HbS polymerization inhibitor that increases the Hb affinity for oxygen by binding to α-globin in a dose-dependent manner to maintain Hb in an oxygenated state and reduce sickling of RBCs (Oksenberg et al., 2016; Metcalf et al., 2017). Previous studies have shown that voxelotor increases Hb, reduces hemolysis, improves RBC deformability, and reduces blood viscosity (Dufu et al., 2018; Rab et al., 2019; Vichinsky et al., 2019). In human subjects, voxelotor increases Hb levels and markedly improves RBC deformability when it is subjected to hypoxia.

Agents that improve red cell rheology, like hydroxyurea (HU), can increase Hb without increasing whole blood viscosity the way a similar Hb rise of unmodified HbS would (Lemonne et al., 2015; Dufu et al., 2018). However, unlike HU, voxelotor transiently modifies the red cell.

This raises the concern that if voxelotor is stopped suddenly, the drug-induced extra Hb would revert to its unmodified state and cause a significant rise in whole blood viscosity, increasing the risk of vaso-occlusive crises (VOC), organ damage, and other clinical complications. This effect could theoretically continue until the drug-induced rise in Hb is eliminated by hemolysis. To investigate this, we treated sickle Townes mice with GBT1118, a voxelotor analog with pharmacokinetic properties in SCD mice that allows it to achieve the degree of Hb modification voxelotor targets clinically (Dufu et al., 2019), and measured changes in whole blood viscosity, HVR, and RBC deformability during and after cessation of treatment.

## Materials and Methods

### Animal Care and Maintenance

Sickle cell mice were purchased from the Jackson Laboratory, Bar Harbor, ME, United States (stock 013071). Animals were maintained and bred in a climate-controlled room under a 14-h light cycle in an animal facility of Baylor College of Medicine. Water and chows were provided *ad libitum*. All animal experiments and procedures were approved by the Baylor College of Medicine IACUC.

### Treatment Protocol

Fifty 8- to 12-week-old Townes HbSS mice were fed control chow (2020 Teklad, Envigo) for 7days. The fifty Townes HbSS mice were then divided into five experimental groups of 10 animals each: (1) control group fed the control chow for an additional week, (2) on treatment group was fed chow containing 4g/kg of GBT1118 for 7days, (3) 24h post-treatment group fed chow containing GBT1118 for 7days and returned to control chow for 24h before data collection, (4) 48h post-treatment group fed chow containing GBT1118 for 7days and returned to control chow for 48h, and (5) 72h post-treatment group fed chow containing GBT1118 for 7days and returned to control chow for 72h.

### Blood Collection

Whole blood was collected in a K2-EDTA tube by terminal retro-orbital bleeding after animal sedation with isoflurane and local anesthesia with tetracaine. Blood was filtered through a 70μm cell strainer to remove clots. All tests were conducted within 4h of collection.

### Rheology

Hb and Hct were measured using an ADVIA 120 Hematology Analyzer (Siemens). Values were adjusted for samples diluted 1:1 in normal saline.

Blood viscosity was measured with a cone and plate viscometer (Brookfield DVII+ with CP40 spindle; AMETEK Brookfield, Middlebrook, MA). 500μl of whole blood at 37°C was measured at a shear rates of 45s^−1^ and 225s^−1^ for 30s each to model venous circulation and arterial circulation, respectively (Lemonne et al., 2015). The HVR, an indicator of RBC oxygen carrying capacity, was calculated by dividing the Hct by the viscosity at both shear rates (Lemonne et al., 2015).

Red blood cell deformability was measured by oxygen gradient ektacytometry (Lorrca, RR Mechatronics, Zwaag, Netherlands). Whole blood at room temperature and normalized to 600×10^6^ RBCs was added to 5ml of polyvinylpyrrolidone. 1.5ml of this solution was injected into the instrument and exposed to varying oxygen concentrations for 30min under a constant shear stress of 30Pa. Three parameters were measured as: EI_max_, the baseline RBC deformability under normal oxygen conditions; EI_min_, the RBC deformability under deoxygenated conditions (ranging from 0 to 20 mmHg O_2_); and PoS, the O_2_ concentration at which a 5% decrease from EI_max_ is observed.

### PK Sample Collection

60μl of total blood was frozen at 80°C for blood-PK analysis from all treatment groups. To prepare samples for plasma-PK, blood was centrifugated at 5000rpm, 4°C and 60μl of plasma was frozen at 80°C. GBT1118 concentrations in blood samples were measured using liquid chromatography-tandem mass spectrometry (LC-MS/MS), as previously described (Dufu et al., 2017).

### Hb Occupancy

Hb occupancy represents the percentage of Hb molecules occupied by GBT1118, estimated as a molar ratio of test compound to Hb concentrations. Percent Hb (%Hb) occupancy was calculated by dividing the concentration of GBT1118 in blood by the concentration of Hb in blood multiplied by 100 (Hutchaleelaha et al., 2019).

### Statistical Analysis

Data analyses and graphical presentations were generated using GraphPad Prism, version 7 (GraphPad Software, Inc., La Jolla, CA). Experimental groups were compared by one-way ANOVA followed by Tukey’s multiple comparison tests to compare group means. Results were considered statistically significant if *p*<0.05.

## Results

**Figure 1:**
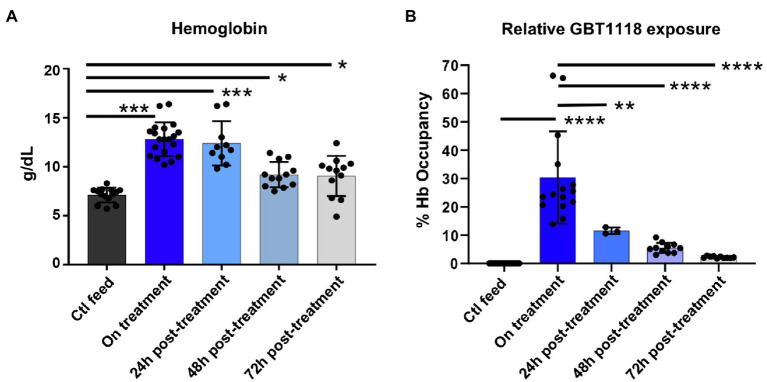
GBT1118 produces Hb rise and drug occupancy comparable to GBT440 in clinical trials. **(A)** Expected rise and fall of total hemoglobin with GBT1118 treatment and its withdrawal. Ctl n=14, on treatment *n*=20, 24h post *n*=10, 48h post *n*=12, and 72h post *n*=12. **(B)** 30% drug occupancy achieved with GBT1118. Ctl n=15, on treatment *n*=20, 24h post *n*=10, 48h post *n*=12, and 72h post *n*=12. ^*^p<0.05; ^**^p=0.051; ^***^p<0.0001; and ^****^p<0.0001.

Mice fed chow containing GBT1118 showed a significant increase in Hb after 1 week. Hb levels dropped rapidly after GBT1118 was withdrawn (Figure 1A). GBT1118 achieved ~30% Hb occupancy, similar to levels observed in clinical trials with voxelotor (Vichinsky et al., 2019), with expected rates of clearance based on the compound half-life (Figure 1B). Because the half-life of GBT1118 in the sickle mouse is 14h, we measured the post-drug effect at least 48h after drug withdrawal.

**Figure 2:**
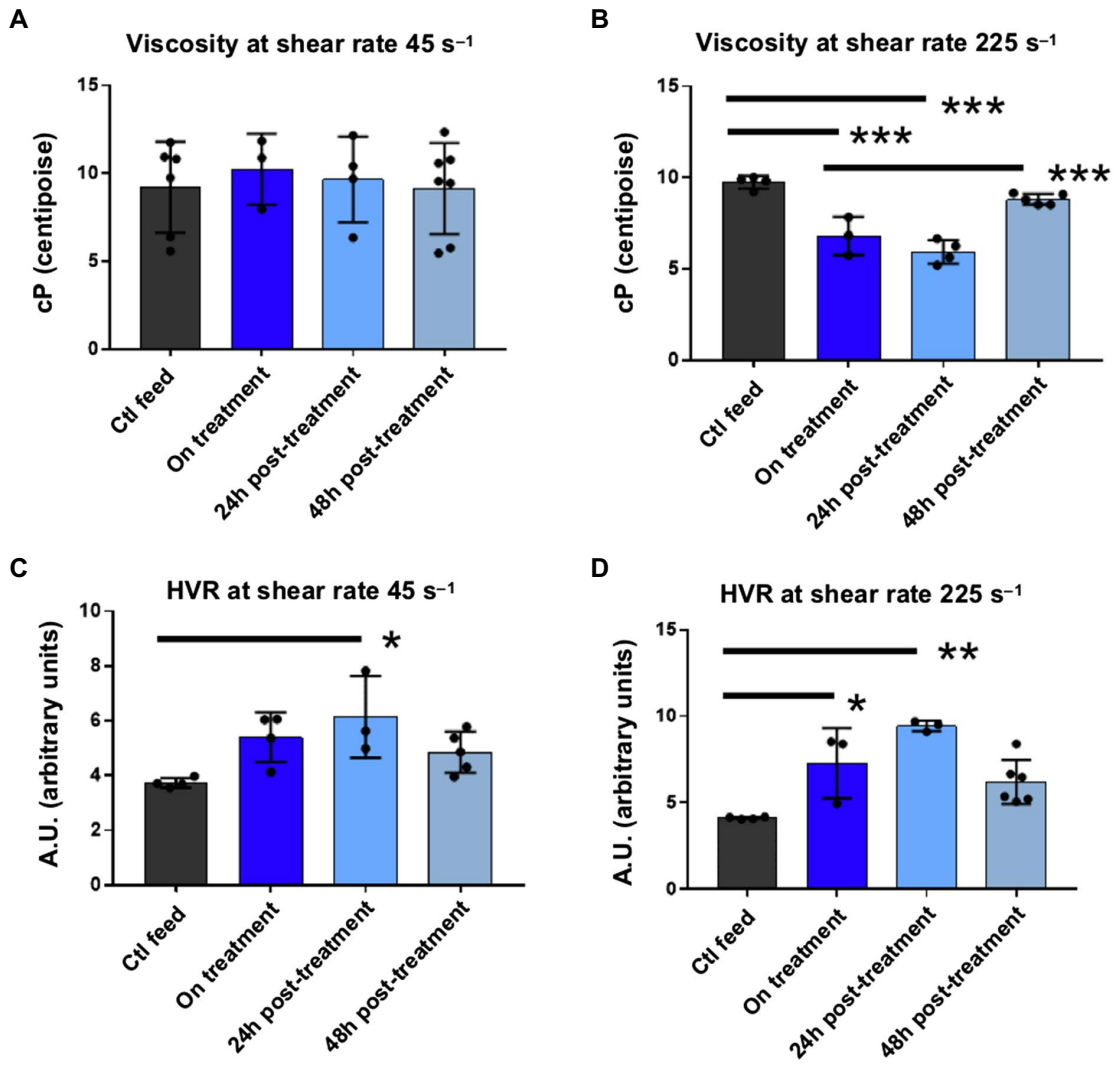
Viscosity and HVR measurements on and off GBT1118. **(A)** At a shear of 45s^−1^, modeling in viscosity from baseline. Ctl n=6, on treatment *n*=3, 24h post *n*=4, and 48h post *n*=7. **(B)** At a shear of 225s^−1^, modeling arterial circulation, viscosity on drug declines from baseline on treatment, and 24h off drug. There is no significant rise in viscosity 48h off drug compared to baseline. Ctl n=4, on treatment *n*=3, 24h post *n*=4, and 48h post *n*=5. **(C)** At an HVR at 45s^−1^, oxygen carrying increased 24h off drug. Ctl n=4, on treatment *n*=4, 24h post *n*=3, and 48h post *n*=5. **(D)** At an HVR at 225s^−1^, oxygen carrying capacity increased on drug and 24h off drug. There was no significant decline in HVR 48h off drug compared to baseline at 225s^−1^ or 45s^−1^ HVR. Ctl *n*=4, on treatment *n*=3, 24h post *n*=3, and 48h post *n*=6. ^***^p<0.0001; ^**^p<0.0051; and ^*^p<0.05. HVR, hematocrit-to-viscosity ratio.

Although Hb rose on drug, viscosity at 45s^−1^ did not changed significantly while on drug (Figure 2A) and declined at 225s^−1^ while on drug (Figure 2B). Withdrawal of drug did not result in a rise in viscosity above baseline values (Figures 2A,B), even at 48h, when most of the drug had been cleared as demonstrated by Hb occupancy (Figure 1B). HVR significantly increased at shear rates 45s^−1^ and 225s^−1^ on drug, suggesting an improvement in oxygen carrying capacity consistent with the known mechanism of GBT1118 (Figures 2C,D). HVR was still elevated 48h off drug compared to control/baseline, but this difference was not statistically significant (Figures 2C,D). Viscosity and HVR 72h after drug withdrawal could not be collected due to instrument malfunction.

**Figure 3:**
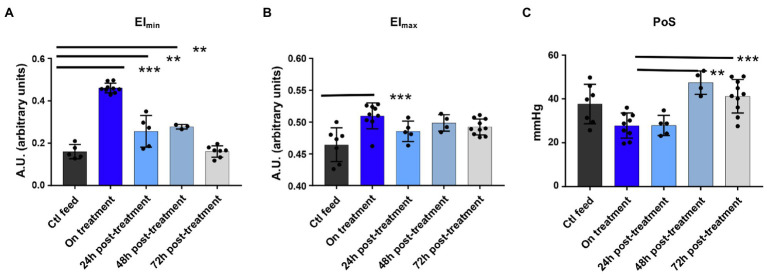
Deformability and point of sickling measurement using oxygen gradient ektacytometry on and off GBT1118. **(A)** RBC deformability when deoxygenated rises on drug and declines but remains above baseline up to 48h off drug. Ctl n=5, on treatment *n*=9, 24h post *n*=5, 48h post *n*=3, and 72h post *n*=7. **(B)** RBC deformability when oxygenated rises on drug. Ctl n=7, on treatment *n*=9, 24h post *n*=5, and 72h post *n*=10. **(C)** Point of sickling drops on drug and returns to baseline 48h drug. Ctl n=7, on treatment *n*=9, 24h post *n*=5, 48h post *n*=4, and 72h post *n*=10. ^***^p<0.0001; ^**^p<0.05. EI_max_, maximum elongation index; EI_min_, minimum elongation index; PoS, point of sickling; and RBC, red blood cell.

GBT1118 fed mice exhibited improved RBC deformability compared to control fed mice. The EI_max_ and EI_min_, deformability under normal and minimal oxygen conditions, both increased. EI_min_ was more improved than EI_max_ (Figures 3A,B), consistent with the mechanism of GBT1118. Point of sickling (PoS), the oxygen concentration at which deformability begins to decline, likely due to polymerization, was reduced in the RBC of animals treated with GBT1118, showing the treated RBCs tolerated a lower oxygen concentration before beginning to sickle. PoS returned to that of control animals 48h after drug cessation (Figure 3C). Interestingly, despite decline in drug occupancy and rise in PoS consistent with drug clearance, the EI_min_ remained elevated above baseline (Figure 3A). Overall, Hb, HVR, and markers of RBC deformability all improved while on treatment. After a 48-h clearance of GBT1118, Hb and EI_min_ were higher in animals fed GBT1118 than control chow.

## Discussion

Whole blood viscosity did not rise on drug despite the rise in Hb. This suggests a qualitative improvement in whole blood, as an increase in sickle Hb without a qualitative improvement would result in a significant viscosity increase (Li et al., 2016). Since viscosity measurements are performed under oxygenated conditions, this improvement is not likely due to the improvement in EI_min_, since this is a measure of RBC deformability under deoxygenated conditions. EI_max_, the oxygenated RBC deformability, was modestly improved. Reduction in hemolysis and the resulting reduction in inflammation may also reduce plasma viscosity. The effect of GBT1118 on red cell density was not directly measured, but there was no change in MCHC to suggest a dense cell reduction. HVR rose on drug, consistent with drug mechanism.

Whole blood viscosity did not rise when GBT1118 was stopped, despite clear decline in PoS, indicating drug was no longer bound to HbS. As GBT1118 maintains Hb in an oxygenated state, the expected rapid rise, or worsening, in PoS was noted with drug dissociation at 48- and 72-h post-treatment. However, the EI_min_ remained elevated/improved after the PoS worsened, indicating drug clearance. These findings may be due to retained improvement in red cell rheology off drug combined with rapid return to baseline Hb levels from hemolysis of sRBCs. A possible mechanistic explanation for persistent red cell quality improvement after drug clearance is as follows: Red cells modified with GBT1118 are less likely to sickle than unmodified RBC. Repeated cycles of hypoxia-driven sickling make RBCs stiffer and increase viscosity (Papageorgiou et al., 2018). These benefits would be expected to be retained for the life of the drug-impacted RBC, not just the duration of drug binding. The residual benefit of improved EI_min_ suggests the RBCs retain an improved deformability under deoxygenated conditions. A recent study showed that 5-hydroxymethyl-2-furfural, an anti-sickling compound, improved longevity of RBCs by reducing mechanical and hypoxia-induced damage (Qiang et al., 2021). This could explain the residual benefits of GBT1118: The RBC had less sickling events which subsequently preserved favorable deformability, and offset the impact of higher Hb on whole blood viscosity. We conclude that concerns regarding viscosity rising above pre-treatment levels upon sudden cessation of voxelotor are not warranted.

In its phase III clinical trial in individuals with SCD, voxelotor was associated with a trend to lower VOC in clinical trials, although not significantly (Howard et al., 2020; Vichinsky et al., 2020). However, the trial was not designed or powered to detect a pain benefit.

Current SCD therapies have similar rheological benefits for individuals with SCD. HU, the most widely used SCD therapy, induces fetal hemoglobin (HbF), a Hb which does not sickle and therefore prevents polymerization of Hb (Rai and Ataga, 2020). HU also reduces white blood cell count, circulating reticulocytes, RBC adhesion, hemolysis, and causes macrocytosis which reduces RBC dehydration and density (Cannas et al., 2017). HU improves sRBC deformability by increasing EI_min_ and EI_max_ and reducing PoS. These functional improvements are only modestly associated with HbF induction; improvements in RBC hydration also contribute (Rab et al., 2019). HU increases Hb, but because the quality of the RBC is improved, viscosity does not rise significantly; therefore, individuals on HU do not pay a viscosity price for their higher Hb and have improved HVR (Li et al., 2016). Placebo-controlled clinical trials in pediatric and adult populations with SCD have shown that HU therapy significantly decreases SCD-related complications like VOC, acute chest syndrome, the need for transfusion, and hospitalizations (Charache et al., 1995; Wang et al., 2011). HU further reduces SCD morbidity by decreasing the risk of stroke and chronic kidney disease. Despite its multiple benefits, a substantial number of SCD patients on HU may not obtain an adequate clinical response. Additional second line agents like voxelotor may benefit patients already on HU.

Blood transfusion and red cell exchange are another therapeutic option that reduces the concentration of circulating sRBCs by introducing normal RBCs, which can decrease the occurrence of complications like VOC and stroke (Estcourt et al., 2020). A common indication for chronic transfusion is an abnormal transcranial Doppler (TCD) ultrasound velocity across the vessels of the circle of Willis, indicating increased risk of stroke, or an overt stroke (Adams et al., 1992, 1998). Hb levels above 9g/dl have been shown to be protective against stroke in SCD (Chou et al., 2020). However, blood transfusions can contribute to other problems common in SCD, such as the development of antibodies to proteins on donor RBCs (alloimmunization), accumulation of too much iron in the body from repeated transfusions, and increased risk of infection. Increasing Hb with voxelotor rather than transfusion will help patients avoid transfusion-related risks of alloimmunization and iron overload, while potentially preventing conversion to abnormal TCD velocity. A clinical trial to investigate this use for voxelotor is underway.

There are several study limitations to note in using the Townes mouse model to study the impact of voxelotor on rheology. Mice were treated with GBT1118 instead of GBT440 (voxelotor) as the former has better PK properties in Townes mice, allowing it to achieve a similar degree of Hb occupancy to voxelotor in clinical use. sRBCs have shorter survival in the Townes mouse model compared to humans with SCD (7days and 20days, respectively). There may be other unknown species or drug-related factors contributing additional differences. Additionally, dense RBCs, RBCs with a Hb concentration >1.11mg/ml, are another valuable rheological biomarker as they are more likely to sickle and contribute to increased blood viscosity. In human subjects, %DRBCs, measured with a phthalate or Percoll gradient, or an ADVIA hematology analyzer, may provide additional information (Ballas and Smith, 1992). However, this value cannot be obtained on mouse RBCs using the ADVIA due to differences in size between mouse and human RBC, and gradients were not used due to blood volume limitations in the mouse model.

Further studies in patients treated with voxelotor may be needed to determine if voxelotor produces additional benefits, such as reduction in oxidative stress, that outlive its presence in the red cell, duration of EI_max_ and EI_min_ elevation after drug clearance, and the compound’s role in reducing VOC and organ damage.

## Data Availability Statement

The raw data supporting the conclusions of this article will be made available by the authors, without undue reservation.

## Ethics Statement

The animal study was reviewed and approved by the Baylor College of Medicine IACUC.

## Author Contributions

DN, MP, DO, and VS designed the study. CK, DN, MP, and DO performed the experiments and analyzed the data. VS supervised the project. CK wrote the manuscript with input from all authors. All authors contributed to the article and approved the submitted version.

## Funding

This work was supported by Global Blood Therapeutics.

## Conflict of Interest

MP – Global Blood Therapeutics: Current Employment, Current equity holder in publicly-traded company. DO – Global Blood Therapeutics: Current Employment, Current equity holder in publicly-traded company. VS – Global Blood Therapeutics: Research Funding.

The remaining authors declare that the research was conducted in the absence of any commercial or financial relationships that could be construed as a potential conflict of interest.

## Publisher’s Note

All claims expressed in this article are solely those of the authors and do not necessarily represent those of their affiliated organizations, or those of the publisher, the editors and the reviewers. Any product that may be evaluated in this article, or claim that may be made by its manufacturer, is not guaranteed or endorsed by the publisher.
